# Erythropoietin Improves the Survival of Fat Tissue after Its Transplantation in Nude Mice

**DOI:** 10.1371/journal.pone.0013986

**Published:** 2010-11-15

**Authors:** Saher Hamed, Dana Egozi, Danny Kruchevsky, Luc Teot, Amos Gilhar, Yehuda Ullmann

**Affiliations:** 1 The Bruce Rappaport Faculty of Medicine, Technion-Israel Institute of Technology, Haifa, Israel; 2 Department of Plastic and Reconstructive Surgery, Rambam Health Care Campus, Haifa, Israel; 3 Department of Plastic and Reconstructive Surgery and Wound Healing, Lapeyronie, Montpellier, France; Leiden University Medical Center, Netherlands

## Abstract

**Background:**

Autologous transplanted fat has a high resorption rate, providing a clinical challenge for the means to reduce it. Erythropoietin (EPO) has non-hematopoietic targets, and we hypothesized that EPO may improve long-term fat graft survival because it has both pro-angiogenic and anti-apoptotic properties. We aimed to determine the effect of EPO on the survival of human fat tissue after its transplantation in nude mice.

**Methodology/Principal Findings:**

Human fat tissue was injected subcutaneously into immunologically-compromised nude mice, and the grafts were then treated with either 20 IU or 100 IU EPO. At the end of the 15-week study period, the extent of angiogenesis, apoptosis, and histology were assessed in the fat grafts. The results were compared to vascular endothelial growth factor (VEGF)-treated and phosphate-buffered saline (PBS)-treated fat grafts. The weight and volume of the EPO-treated grafts were higher than those of the PBS-treated grafts, whose weights and volumes were not different from those of the VEGF-treated grafts. EPO treatment also increased the expression of angiogenic factors and microvascular density, and reduced inflammation and apoptosis in a dose-dependent manner in the fat grafts.

**Conclusions/Significance:**

Our data suggest that stimulation of angiogenesis by a cluster of angiogenic factors and decreased fat cell apoptosis account for potential mechanisms that underlie the improved long-term survival of fat transplants following EPO treatment.

## Introduction

During angiogenesis, endothelial cells can produce proteases such as matrix metalloproteinases (MMPs), and can increase their ability to migrate and proliferate [1]. This process depends on the activity of several growth factors, such as vascular endothelial growth factor (VEGF), basic fibroblast growth factor (bFGF) and platelet-derived growth factor (PDGF)-BB [2], [3], [4].

Erythropoietin (EPO), a glycoprotein hormone that stimulates erythropoiesis, also instigates the secretion of angiogenic factors [5], [6]. Ribatti and colleagues demonstrated that EPO induced a pro-angiogenic phenotype in cultured endothelial cells, and stimulated angiogenesis in vivo [7], [8]. It also stimulated angiogenesis indirectly in ischemic tissue by increasing the expression of VEGF and by recruiting endothelial progenitor cells [9], [10]. In rats, EPO administration mobilized bone marrow-derived progenitor cells [11] and increased the myocardial expression of VEGF [12]. Wang *et al.* demonstrated that EPO can promote angiogenesis by stimulating VEGF secretion from neural progenitor cells and VEGF-receptor expression in cerebral endothelial cells [13].

Other non-hematopoietic effects of EPO include cytoprotection of vascular endothelial cells [14], [15] and anti-apoptotic actions in vascular smooth muscle cells and endothelial cells [16] such as prevention of mitochondrial release of cytochrome c, suppression of caspase activity, and upregulation of the activity of the protein kinase B (PKB) signaling pathway and the expression of the antiapoptotic protein Bcl-xl [17], [18].

Autologous fat transplantation is a common and ideal technique for soft tissue augmentation and for filling soft tissue defects due to trauma or aging [19]. Emerging evidence suggests that early and adequate vascularization of the fat graft is essential for its take and viability [20], [21]. However, the relatively high resorption rate of the fat graft reduces the efficacy of this technique because the volume of vascularized grafts continues to decline as a result of increased fat cell death after its transplantation [22]. Although angiogenic factors [23], [24], and VEGF gene therapy, have been individually used to stimulate angiogenesis in fat grafts in order to enhance fat cell survival and viability [21], [25], [26], the clinical outcome has been disappointing, because a single angiogenic factor to stimulate angiogenesis may be inadequate [27]. Therefore, reducing the resorption rate of transplanted fat is a clinical challenge.

In light of all these findings, we hypothesized that treatment of fat grafts with EPO would (a) stimulate the release of several angiogenic factors and promote angiogenesis, and (b) prevent apoptosis in fat grafts. Using this working hypothesis, we initiated a study whose aims were (a) to evaluate and compare the effects of VEGF and EPO on fat cell survival and angiogenesis in human transplanted fat tissue, and (b) to investigate the long-term survival of grafted fat cells after EPO treatment in immunologically-compromised nude mice.

## Materials and Methods

### Isolation and preparation of human fat tissue

Fat was harvested from the thigh of a 40-year-old woman undergoing suction-assisted lipectomy under general anesthesia. In order to decrease bleeding during fat aspiration, and to relieve pain after the procedure, the areas for aspiration were injected with a local anesthesia solution containing lidocaine (0.5%) and adrenaline (1∶1,000,000) before the beginning of the procedure. The fat was aspirated using a 14-gauge three-hole blunt cannula, and then processed under sterile conditions for subsequent grafting into nude mice within two hours of its collection according to previously published protocols [28], [29]. The participant gave her written informed consent, and the study was reviewed and approved by the institutional review board of the Rambam Health Care Campus.

### Study design

Two different animal studies were conducted, and the use of animals and all the experimental procedures were reviewed and approved by the Technion Animal Care and Use Committee. The first study comprised 30 seven-week-old female CD-1 nude mice (Harlan, Jerusalem, Israel), which were housed in cages in a room with an artificial 12-h light/dark cycle at a constant temperature range (24±2°C) and relative humidity (55±10%). The mice were acclimated for one week prior to the study, and fed a standard chow and water ad libitum. The 30 mice were randomly divided into three equal groups according to treatment of the aspirated human fat after its injection. Group 1 mice were injected with 1 ml of human fat that was treated with sterile phosphate buffer saline (PBS) (control group). Group 2 mice were injected with 1 ml human fat that was treated with 1000 IU/kg EPO (low-dose EPO group). Group 3 mice were injected with 1 ml human fat that was treated with 5000 IU/kg EPO (high-dose EPO group). The fat was injected subcutaneously into the scalp using a 14G needle while the animals were manually restrained. Immediately following fat transplantation, the fat grafts were injected with 100 µl PBS (control group), or with either 20 IU EPO/100 µl PBS (low-dose EPO group) or 100 IU EPO/100 µl PBS (high-dose EPO group) every three days for 18 days making a total of 6 equal injections of each treatment per fat graft. EPO was purchased as an injection ampoule (ARANESP®, Amgen AG, Zug, Switzerland) which contained 150 µg/ml (18,000 IU) of EPO.

The second animal experiment used 20 seven-week-old female CD-1 nude mice, and differed from the first experiment in that the fat grafts were treated with PBS or VEGF (2 μg/ml) (Sigma Aldrich, MO, USA) after its injection into the 20 mice. Briefly, 100 µl PBS or 200 ng VEGF/100 µl PBS were injected every three days for 18 days in an identical manner to that of the PBS- or EPO-treated mice in the first experiment. The PBS-treated mice in the second experiment were used as a second control group. Post-operative analgesics and antibiotics were not administered to the mice in the two experiments.

### Follow-up and data collection

The duration of the study period of each experiment was 15 weeks after fat transplantation. On the day of fat injection, 18 days after the fat injection, and at the end of the study period, each mouse was weighed, and a tail vein blood sample was collected for determining the red blood cell, leukocyte, and platelet counts, the plasma hemoglobin, VEGF, and EPO concentrations. VEGF and EPO concentrations were determined in the plasma as well as in the homogenates of the samples of the fat grafts using commercial enzyme-linked immunosorbent assays (Quantikine® VEGF immunoassay Kit and Quantikine® IVD® Erythropoietin Kit, R&D Systems, MN, USA) in accordance with the manufacturer's instructions.

**Figure 1:**
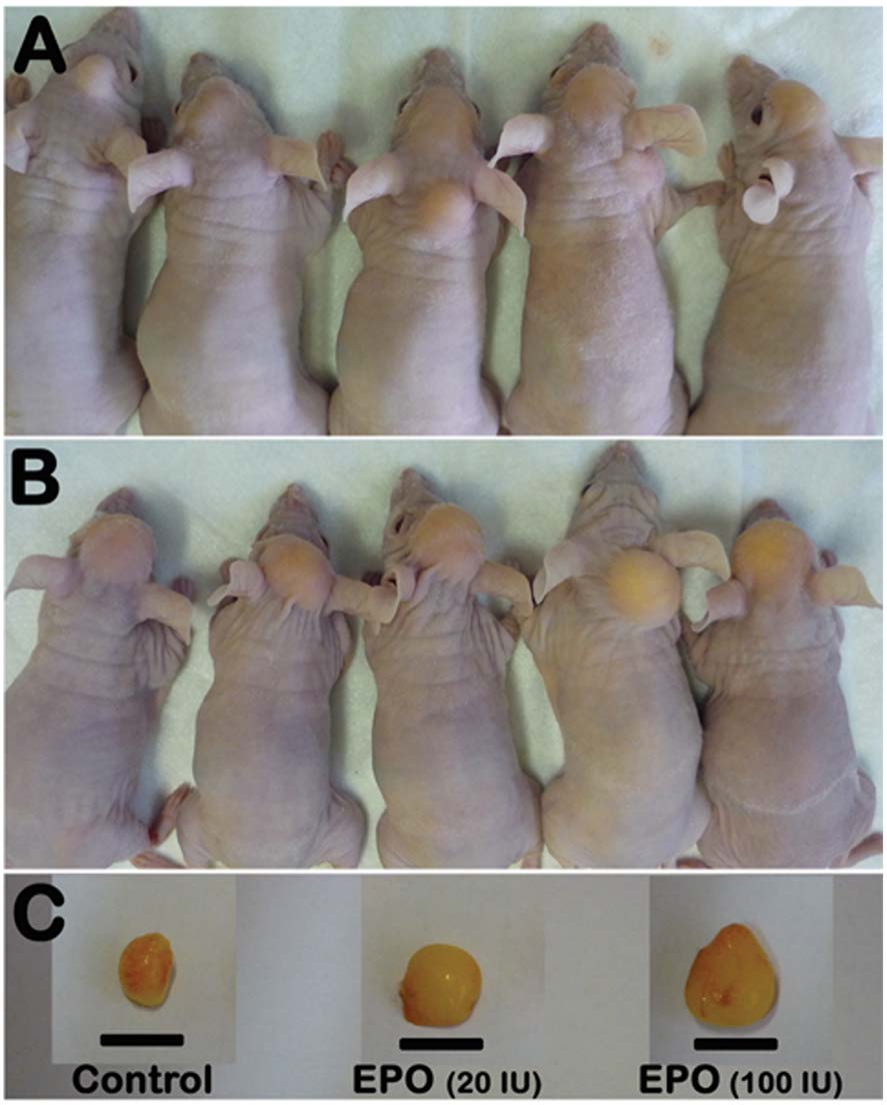
Photograph of five representative mice with fat grafts at the end of the 15-week study period. (A) Five PBS-treated fat grafts with small lumps that vary in size in the scalps. (B) Five high-dose erythropoietin (100 IU EPO)-treated fat grafts with large lumps that are similar in size in the scalps. (C) Fat grafts were dissected from the mice 15 weeks after transplantation. From left to right, a representative small fat graft from a PBS-treated fat graft, an intermediate-size low-dose EPO-treated fat graft, and a large high-dose EPO-treated fat graft respectively. Scale bar: 10 mm.

After 15 weeks, all mice were humanely killed, and the fat grafts were carefully dissected from their scalps (Figure 1C). Each fat graft was weighed, and the volume of the fat graft was measured using the liquid overflow method [30]. After weight and volume determination, each fat graft was divided into two equal portions. One portion was stored at −80°C and used to determine its EPO concentrations, VEGF content, extent of apoptosis, and the expression levels of its angiogenic factors, namely bFGF, insulin growth factor-1 (IGF-1), PDGF-BB, VEGF receptor-2 (VEGFR-2), EPO receptor (EPOR), and MMP-2, the survival factor PKB and phosphorylated PKB, and pro-apoptotic factors, namely caspase 3 and cytochrome c. The second portion was placed in 4% formalin and used for histological examination and for determination of macrophage infiltration, microvascular density (MVD), VEGFR-2 and EPOR localization.

### Histology and immunohistochemistry

Histological slides of the formalin-maintained samples were prepared, and then stained with hematoxylin and eosin using standard procedures. Immunohistochemistry was performed using rabbit monoclonal antibodies against tissue CD31, VEGFR-2 and EPOR, and goat polyclonal IgG against VEGF (R&D Systems, Minneapolis MN, USA), and CD68 (Dako, Glostrup, Denmark). The paraffin-embedded fat graft sections were incubated with the antibodies overnight at room temperature followed by incubation with appropriate secondary antibodies [31]. Upon completion of the incubations, the specimens were counterstained with hematoxylin. Mouse IgG was used as a negative control. The slides were examined under a light microscope for (a) the extent of integration, as evidenced by the extent of organization of intact and nucleated fat cells, (b) the extent of fibrosis, as evidenced by the amount of collagen and elastic fibrils, (c) the presence of cysts and vacuoles, and (d) the intensity of the inflammatory response, as evidenced by the extent of lymphocyte and macrophage infiltration. Each criterion was graded on a scale of 0 to 5 where 0 =  absence, 1 =  minimal presence, 2 =  minimal to moderate presence, 3 =  moderate presence, 4 =  moderate to extensive presence, and 5 =  extensive presence.

Quantification of macrophage infiltration in the fat grafts was estimated by counting the number of CD68-positive cells in five fields per fat graft in all fat graft sections. Microvascular density (MVD) in fat grafts was determined in five regions of interest where the CD31 antibody signal was the most intense in each section in all of the fat graft sections. The number of macrophages and blood vessels in each region was counted under a light microscope at 400× magnification. The assessment in each fat graft was made by calculating the mean result in two different sections per fat graft and five different fields of view per section. All evaluations were made by SH, DK, DE, and YU, who were blind to the treatment of the mice.

### Determination of the extent of apoptosis in the fat grafts

The extent of apoptosis in all fat grafts was assessed by the terminal deoxyuridine triphosphate nick end labeling (TUNEL) assay using a commercial kit (ApopTag® Plus Fluorescein Kit, CHEMICON, CA, USA), in accordance with the manufacturer's instructions. Duplicate determinations were done in each sample, and were processed by fluorescence-activated cell sorting (FACS) (Becton Dickinson, NJ, USA). Data were analyzed using the Macintosh CELLQuest software program (Becton Dickinson).

### In vitro tube formation of HUVECs on matrigel

The *in vitro* angiogenic potential of VEGF and EPO was assessed by their ability to form tubes of endothelial cells on matrigel. To this end, human umbilical vein endothelial cells (HUVECs) were first cultured on fibronectin-coated 6-well plates in endothelial basal medium-2 (EBM-2) (PromoCell, USA) and then treated with 0, 20 or 100 IU/ml EPO for 48 hours before their use in the assay. In a second experiment, HUVECs were exposed to 0, 100 IU/ml EPO and 200 ng/ml VEGF for 48 hours in EBM-2 that contained or lacked 0.25 mg/ml bevacizumab (Avastin®, Genentech, San Francisco, CA, USA), a humanized monoclonal antibody that antagonizes the actions of VEGF. After 48 hours, the untreated HUVECs, the VEGF- and EPO-treated HUVECs, and the VEGF+bevacizumab- and EPO+bevacizumab-treated HUVECs were detached gently by 0.5% trypsin/EDTA, and then suspended in EBM-2. At the same time, frozen matrigel (Sigma Aldrich, St Louis MO, USA) was thawed, and spread onto 96-well plates (40µl/well) at room temperature for 30 minutes to allow solidification. The detached untreated HUVECs, VEGF- and EPO-treated HUVECs, and VEGF+bevacizumab- and EPO+bevacizumab-treated HUVECs (5×10^4^ cells/150µl EBM-2/well) were placed on the matrigel surface, and then incubated at 37°C for 24 hours in EBM-2. After plating on the matrigel, the VEGF- and EPO-treated HUVECs and VEGF+bevacizumab- and EPO+bevacizumab-treated HUVECs were treated a second time with identical concentrations of EPO, VEGF, and bevacizumab, respectively. After 24 hours, the non-integrated cells were removed by washing, and tube formation on the matrigel was assessed under a light microscope at 10× magnification. The tubular structures were graded semiquantitatively by evaluating the presence and stages of tube formation on a scale of 0 to 5: 0 =  well separated individual cells, 1 =  cells had begun to migrate and align themselves, 2 =  visible capillary tubes and no sprouting, 3 =  visible sprouting of new capillary tubes, 4 =  early formation of closed polygons, 5 =  development of complex mesh-like structures. All evaluations were assessed by SH, DE and DK, who were blind to the treatments. Four random high-power fields in each sample were examined from three independent experiments. The results from each examiner were then pooled in order to calculate the mean value for each criterion for each sample in each group.

### HUVEC proliferation

To investigate EPO-induced angiogenesis through mechanisms involving pro-angiogenic factors, we measured the proliferation of EPO-treated HUVECs in the presence of various pro-angiogenic factor inhibitors. To this end, HUVECs (2×10^5^ cells/well) were cultured on fibronectin-coated 12-well plates in EBM-2. The cultured HUVECs were treated with or without 100 IU/ml EPO for 48 hours, and then exposed for 3 hours to (a) 0.25 mg/ml bevacizumab, (b) 100 nM of PD173074; an inhibitor of bFGF (Calbiochem, San Diego, CA), (c) 20 µM of tyrphostin AG 1296; a selective inhibitor of PDGF (sigma), (d) a combination of bevacizumab, PD173074 and tyrphostin, and to (e) 100 nM wortmannin; a phosphatidylinositol 3-kinaz (PI 3-K) inhibitor (sigma). Upon the completion of the experiment, the cells were washed with PBS and then incubated with [^3^H]-thymidine (NEN, Boston, MA, USA) (1 µCi/ml medium) for 5 h at 37°C. Thereafter, 0.5 ml cold 10% Trichloroacetic acid (TCA) was added into each well for another 30 min at 4°C. To extract the ^3^H-thymidine labeled DNA, 0.5 ml 1N NaOH was added to each well for 10 min at room temperature, and then 0.5 ml 1N HCl was added and mixed well. Samples of mixture solution (0.5 ml) was taken from each well and added to scintillation vials for the measurement of [^3^H]-thymidine incorporation to DNA (cpm/mg protein). Duplicate cell counts were averaged for 3 experiments. Data were expressed as the percentage of control.

### Western analysis

The expression levels of the angiogenic factors, bFGF, IGF-1, PDGF-BB, VEGFR-2, EPOR and MMP-2, the cell survival factor PKB and phosphorylated PKB, and the pro-apoptotic factors caspase 3 and cytochrome c were determined in homogenates of the harvested fat grafts using western blot analysis. Homogenates of samples from the fat grafts were lysed in RIPA buffer (R&D Systems). A 40-µg aliquot of each lysate was loaded onto SDS-PAGE, and then transferred to nitrocellulose membranes. Membranes were then incubated with monoclonal antibodies against bFGF, IGF-1, PDGF-BB, MMP-2, PKB, phosphoPKB, caspase 3, and cytochrome c (all purchased from Santa Cruz Biotechnology, Santa Cruz, CA, USA) and with monoclonal antibodies against VEGFR-2 and EPOR (R&D systems), followed by a second incubation with a horseradish peroxidase (HRP)-conjugated IgG secondary antibody (Santa Cruz Biotechnology). An antibody against β-actin (Santa Cruz) was used to normalize protein loading. The resultant bands were quantified by densitometry.

### Statistical analysis of the data

The data for each study parameter from the PBS-, the VEGF- and the EPO-treated fat grafts in each treatment group were pooled, and the results are presented as mean ± standard deviation (SD). The data have a normal distribution by the Kolmogorov-Smirnov test. The data from the first experiment were analyzed by ANOVA and the data from the second experiment were analyzed by Student's *t* test, using a computerized statistical software program (Prism version 5.0, GraphPad Software Inc, CA, USA). Differences were considered statistically significant when *P*≤0.05. Kappa values for intra-examiner repeatability of the blinded evaluations of histological analysis, MVD, and tube formation on matrigel were 0.94, 0.89, and 0.93, respectively.

### Results

All mice in all of the treatment groups of both experiments completed the 15-week study period. They appeared to be healthy and there was no evidence of cachexia during the entire study period. There were no significant changes in red blood cell, leukocyte, and platelet counts, and in plasma hemoglobin and EPO concentrations in the mice that had either PBS-treated- or low-dose EPO-treated fat grafts (Table 1). The red blood cell, leukocyte platelet counts, and plasma EPO concentrations, but not the plasma hemoglobin concentrations, were significantly increased in the mice with high-dose EPO-treated fat grafts (Table 1). Eighteen days after transplantation, plasma VEGF concentrations were significantly increased in both groups of mice with EPO-treated fat grafts. At the end of the 15-week study period, the plasma VEGF concentrations in the two groups of mice with EPO-treated grafts were not significantly different from baseline values, or from those in mice with PBS-treated fat grafts. EPO concentrations in the PBS- and EPO-treated grafts were not different from each other at each of the three time points (Table 1).

**Table 1:** Effect of EPO treatment on body weight, hematology, and plasma and tissue EPO concentrations in the three experimental groups.

Group	Control (n = 10)	Low-dose EPO (n = 10)	High-dose EPO (n = 10)
**Initial mice weight ** ***(g)***	26.7±1.1	25.9±1.1	26.2±1.0
After EPO treatment	27.3±1.1	27.9±1.1	28.6±1.2
At week 15	28.3±1.1	28.8±1.1	29.0±1.2
**Initial RBC count ** ***(10^6^/mm^3^)***	7.8±0.9	8.0±1.0	7.9±1.2
After EPO treatment	7.9±0.9	8.0±1.1	8.9±1.0*
At week 15	7.8±0.9	7.9±1.0	8.1±1.2
**Initial leukocyte count ** ***(10^6^/mm^3^)***	10.8±1.2	11.1±1.1	10.9±1.2
After EPO treatment	11.2±1.2	11.4±1.1	13.1±1.3*
At week 15	11.0±1.1	10.8±1.1	11.4±1.2
**Initial platelet count ** ***(10^3^/L)***	593±54	609±63	603±72
After EPO treatment	579±58	621±68	741±81**
At week 15	593±54	601±57	597±64
**Initial hemoglobin conc. ** ***(g/dl)***	14.4±1.3	15.1±1.4	15.5±1.4
After EPO treatment	14.8±1.3	15.7±1.4	16.4±1.6
At week 15	14.8±1.2	15.1±1.6	14.9±1.5
**Initial plasma EPO conc. ** ***(mU/mL)***	14.3±1.9	14.6±1.3	14.2±1.7
After EPO treatment	13.7±1.4	17.6±3.3*	46.7±8.7***
At week 15	14.3±1.7	14.2±1.3	14.1±1.3
**Initial plasma VEGF conc. ** ***(pg/mL)***	38.6±3.9	34.8±4.6	39.2±4.8
After EPO treatment	37.1±3.8	51.5±6.6*	87±9.2***
At week 15	38.0±3.3	36.6±4.9	37.4±5.3
**Tissue EPO conc. ** ***(mU/mL)***	0.3±0.1	0.3±0.1	0.3±0.1

**Footnotes**

Values are presented as mean ± SD; n  =  number of mice; conc.  =  concentrations; RBC  =  red blood cells; EPO  =  erythropoietin; VEGF  =  vascular endothelial growth factor. *P<0.05, **P<0.01, ***P<0.001 for the difference between either the low-dose- or the high-dose-treated EPO grafts and the PBS-treated grafts.

### Fat graft weights and volumes

A well-defined subcutaneous lump was observed on the scalp of each mouse at the end of the 15-week study period (Figure 1). The weights and volumes of the EPO-treated grafts were higher than those of the PBS-treated grafts (Table 2). The weights and volumes of the PBS-treated fat grafts in the first experiment were not different from those of the PBS- and VEGF-treated grafts in the second experiment (Table 2).

**Table 2:** Effect of EPO treatment on fat graft weight and volume in all treatment groups in the two experiments.

	First experiment			Second experiment	
Group	PBS(n = 10)	Low-doseEPO(n = 10)	High-doseEPO(n = 10)	PBS(n = 10)	VEGF(n = 10)
**Weight ** ***(g)***	0.3±0.1	0.5±0.2**	0.6±0.2***	0.32±0.2	0.35±0.2
**Volume ** ***(ml)***	0.3±0.1	0.4±0.1**	0.6±0.1***	0.35±0.1	0.36±0.2

**Footnotes**

Values are presented as mean ± SD.

n  =  number of mice.

EPO  =  erythropoietin.

VEGF  =  vascular endothelial growth factor.

**P<0.01, ***P<0.001, for the difference between either the low-dose- or the high-dose EPO-treated fat grafts and the PBS-treated grafts.

### Histological evaluation

**Figure 2:**
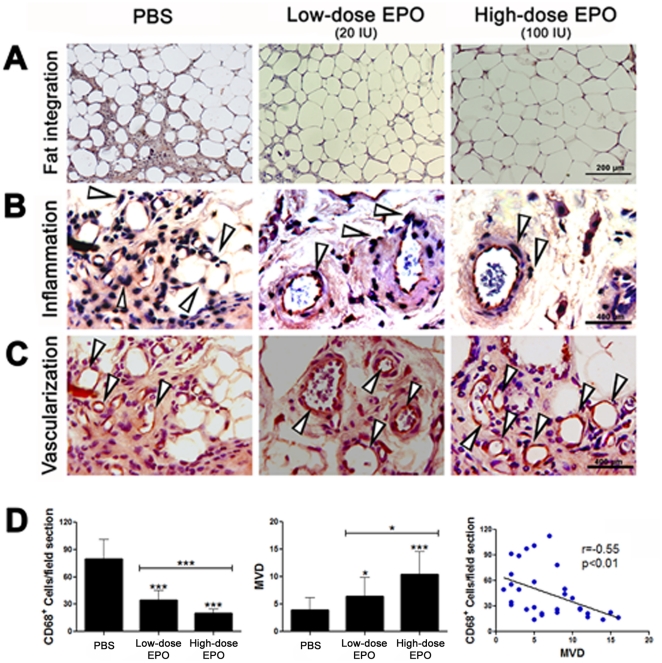
Histological sections of fat grafts that were removed from the PBS-treated, low-dose, and high-dose EPO treated mice 15 week after fat transplantation. The fat grafts from three different groups of mice were treated with either PBS (100 µl), 20 IU EPO/100 µl PBS (low-dose), or 100 IU EPO/100 µl PBS (high-dose) on the day of the fat injection, and the treatments were repeated every three days for 18 days. After harvesting, sections were stained with hematoxylin and eosin or prepared for assessing inflammatory cell infiltration and MVD, and then examined under a light microscope for (a) the extent of integration, as evidenced by the extent of organization of intact and nucleated fat cells in the grafted fat tissue architecture, (b) the extent of fibrosis, as evidenced by the amount of collagen and elastic fibrils, (c) the presence of cysts and vacuoles, (d) the intensity of the inflammatory response, as evidenced by the extent of macrophage infiltration, and (e) MVD, as evidenced by the number of blood vessels in the fat grafts. Representative histological micrographs of PBS-, and low-dose- and high-dose-EPO treated fat grafts (left to right). (A) fat cell integration in fat grafts, (B) inflammation as evidenced by infiltration of CD68-positive cells (macrophages) in fat grafts, and (C) vascularization in the fat grafts as evidenced by MVD which was quantified by counting CD31-positive vessels. The arrows are pointing to dark-stained CD68-positive macrophages and to red-stained CD31-positive endothelial cells. (D) EPO treatment decreases inflammation (left) and increases MVD (middle) in a dose-dependent manner that correlates negatively (right). Each bar represents the mean CD68-positive cells or MVD ± SD from five regions of interest in each fat graft from each treatment group at the end of the 15-week study period. *P<0.05, ***P<0.001, for the difference between either the low-dose- or the high-dose EPO-treated fat grafts and the PBS-treated grafts. Scale bar for A: 200µm, scale bar for B or C: 400µm.

The histological criteria of the PBS-treated fat grafts in the first experiment were not different from those in the second experiment. The extent of integration of the fat graft was higher in the high-dose EPO-treated grafts than in the low-dose EPO- and PBS-treated grafts (Figure 2A), and, the extent of cyst formation and fibrosis was lower in the high-dose EPO-treated grafts than in the low-dose EPO- and PBS-treated grafts (Table 3). The extent of integration, cyst formation, and fibrosis in the VEGF-treated grafts were not different from those in the PBS-treated grafts (Table 3).

**Table 3:** Histological analysis of the dissected fat grafts in all treatment groups in the two experiments.

	First experiment			Second experiment	
Group	PBS(n = 10)	Low-doseEPO(n = 10)	High-doseEPO(n = 10)	PBS(n = 10)	VEGF(n = 10)
**Integration**	3.3±1.0	4.3±0.8	4.6±0.7*	3.6±0.7	3.2±0.9
**Fibrosis**	2.5±0.9	2.1±0.6	1.5±0.7*	2.6±0.5	2.9±0.7
**Cyst/Vacuoles**	2.8±0.9	2.0±0.9	1.7±0.7*	2.9±1.0	3.3±1.0
**Inflammation**	2.9±1.1	1.7±0.5*	1.3±0.6**	3.2.0±1.4	4.0±1.2*

**Footnotes**

Values are presented as mean ± SD.

n  =  number of mice.

EPO  =  erythropoietin.

VEGF  =  vascular endothelial growth factor.

*P<0.05, **P<0.01 for the difference between either the low-dose- or the high-dose EPO-treated fat grafts and the PBS-treated grafts.

### The effect of EPO on inflammatory response and MVD in the fat grafts

The severity of the inflammatory response as evidenced by CD68-positive cells infiltration in fat grafts both in the low-dose and in the high-dose EPO-treated fat grafts was lower than the severity of the inflammatory response in the PBS-treated fat grafts. The severity of the inflammatory response in the high-dose EPO-treated grafts was significantly lower than that observed in the low-dose EPO-treated grafts (Figure 2B and 2D left). However, the intensity of the inflammatory response in the VEGF-treated fat grafts was significantly higher than that observed in the PBS-treated fat grafts (Table 3).

The MVDs observed in both the high-dose and low-dose EPO-treated fat grafts were significantly higher than the MVDs of the PBS-treated fat grafts, and the effect of EPO on MVD was dose-dependent. Avascular areas, ectatic vessels with edema and perivascular hemorrhage, and a marked reduction in capillary ramification were observed in the PBS-treated fat grafts. In the EPO-treated fat grafts, there were well-vascularized areas with increased expression of CD31, and numerous endothelial islets (Figure 2C and 2D middle). The extent of MVD was negatively correlated to the extent of macrophage infiltration in the fat grafts (Figure 2D right).

### The effect of EPO on VEGF content and expression levels of angiogenic factors and PKB in the fat grafts

**Figure 3:**
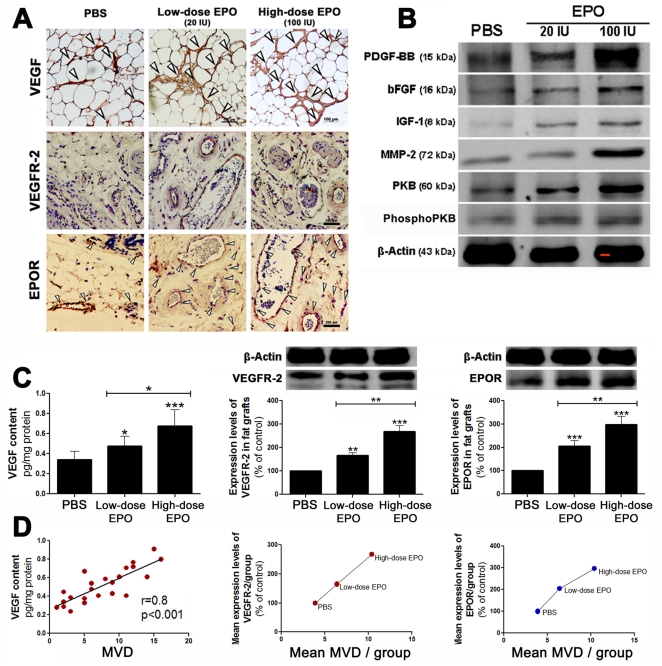
Effect of EPO on the expression levels of angiogenic growth factors in the fat grafts. The fat grafts from the three different groups of mice were treated with either PBS (100 µl), 20 IU EPO/100 µl PBS (low-dose), or 100 IU EPO/100 µl PBS (high-dose) on the day of the fat injection, and the treatments were repeated every three days for 18 days. (A) Representative histological micrographs of PBS-, and low-dose- and high-dose-EPO treated fat grafts (left to right) presenting VEGF expression (upper panel), VEGFR-2 expression (middle panel), and EPOR expression (lower panel). (B) Representative western blots of the expression levels of the angiogenic factors in the PBS- and EPO-treated fat grafts at the end of the 15-week study period. bFGF: basic fibroblast growth factor; IGF-1: insulin-like growth factor-1; PDGF-BB: platelet-derived growth factor-BB; MMP-2: matrix metalloproteinase-2; PKB: protein kinase B; phosphoPKB: phosphorylated PKB. (C) Graphs representing the mean VEGF content (left), the mean VEGFR-2 expression (middle) and the mean EPOR expression (right) ± SD in the fat grafts in each treatment group. (D) The correlation between VEGF and MVD (left), and between mean VEGFR-2 (middle) and EPOR (right) expression and mean MVD in each group. *P<0.05, **P<0.01, ***P<0.001for the difference between either the low-dose- or the high-dose EPO-treated fat grafts and the PBS-treated grafts. Scale bar: 200µm.

The VEGF contents in the low-dose and high-dose EPO-treated fat grafts were significantly higher than the VEGF contents in the PBS-treated fat grafts. The VEGF content in the high-dose EPO-treated grafts was significantly higher than that observed in the low-dose EPO-treated graft (Figure 3A upper panel and 3C left). EPO induced a dose-dependent increase in the expression levels of bFGF, IGF-1, PDGF-BB, MMP-2, PKB, and phosphoPKB (Figure 3B). Furthermore, EPO increased both tissue VEGFR-2 and EPOR expression in a dose-dependent manner, as evidenced by immunohistochemical localization of both factors (Figure 3A middle and lower panels respectively) and by western blot analysis (Figure 3C middle and left respectively). The VEGF content and the mean expression levels of both VEGFR-2 and EPOR were positively correlated with MVD (Figure 3D).

### The effect of EPO on the extent of apoptosis in the fat grafts

**Figure 4:**
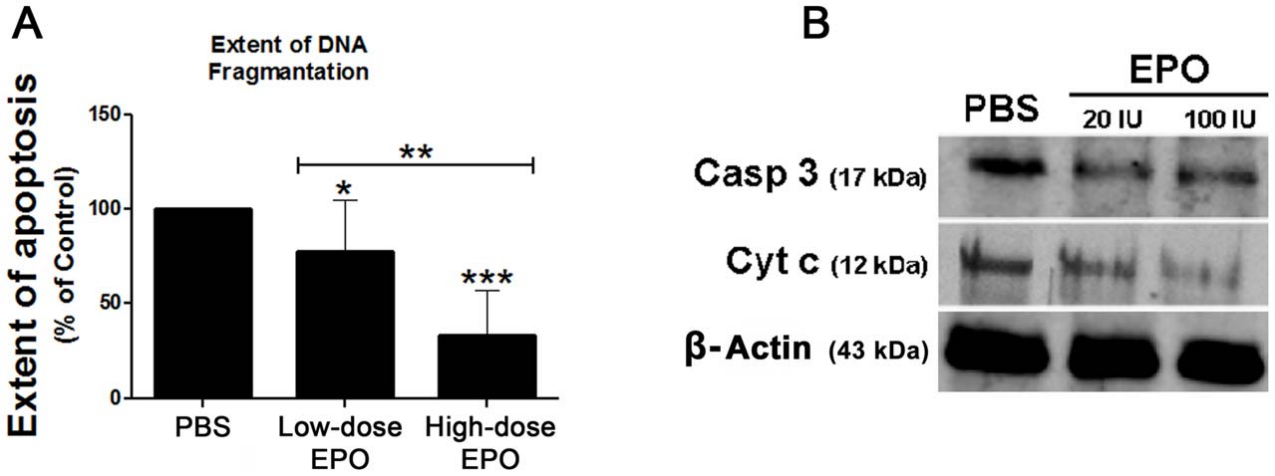
Effect of EPO on the extent of apoptosis in the fat grafts. PBS (100 µl), 20 IU EPO/100 µl PBS (low-dose), or 100 IU EPO/100 µl PBS (high-dose) were injected into fat grafts in three different groups of mice on the day of the fat injection and then repeated every three days for 18 days. (A) The extent of apoptosis was measured by the TUNEL assay, and is expressed as a percentage of the presence of apoptosis in the PBS-treated fat grafts. Each bar represents the mean extent of apoptosis ± SD in the fat graft in each treatment group at the end of the 15-week study period. *P<0.05, **P<0.01, ***P<0.001 for the difference between either the low-dose- or the high-dose EPO-treated fat grafts and the PBS-treated grafts. (B) Representative western blots of the expression levels of caspase 3 (Casp 3) and cytochrome c (Cyt c) in the PBS- and EPO-treated fat grafts at the end of the 15-week study period.

The extent of apoptosis in the PBS-treated fat grafts was greater than that in the low-dose and high-dose EPO-treated fat grafts. The extent of apoptosis in the high-dose EPO-treated fat grafts was significantly lower than that in the low-dose EPO-treated graft (Figure 4A). EPO caused a dose-dependent decrease in the expression levels of caspase 3 and cytochrome c (Figure 4B).

### The effect of VEGF on MVD and extent of apoptosis in the fat grafts

**Figure 5:**
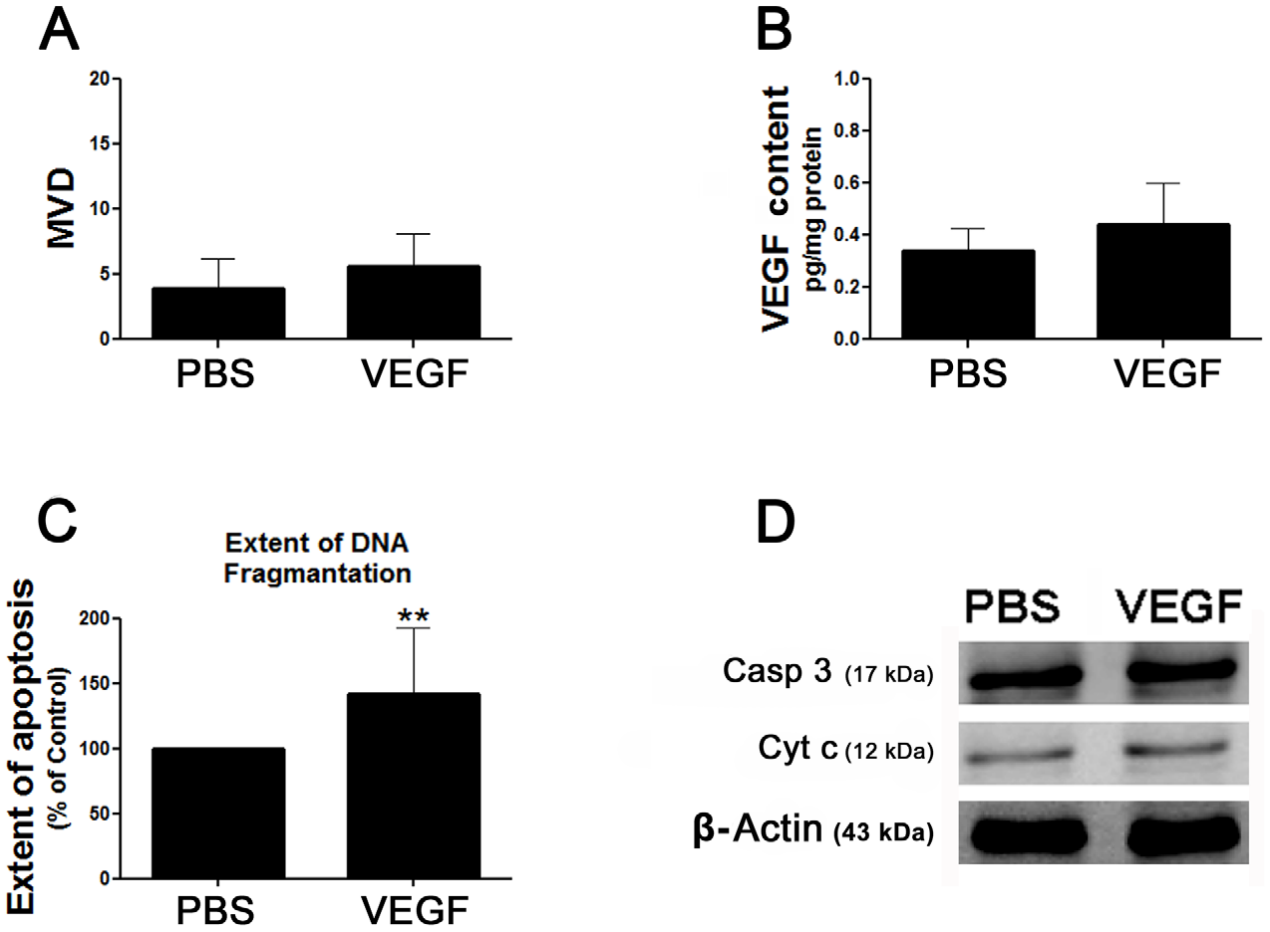
Effect of VEGF on MVD and the extent of apoptosis in the fat grafts. PBS (100 µl) or VEGF (200 ng VEGF/100 µl PBS) were injected into the fat grafts in two different groups of mice on the day of the fat injection and every three days for 18 days. (A) Each bar represents the mean MVD ± SD from five regions of interest in each slide that was prepared from the harvested fat grafts of each treatment group at the end of the 15-week study period. (B) Each bar represents the mean VEGF content ± SD in the harvested fat grafts in each treatment group at the end of the 15-week study period. (C) The extent of apoptosis was measured by the TUNEL assay, and is expressed as a percentage of the extent of apoptosis in the PBS-treated fat grafts. Each bar represents the mean extent of apoptosis ± SD in the fat graft in each treatment group at the end of the 15-week study period. **P<0.01for the difference between the VEGF-treated fat grafts and the PBS-treated grafts. (D) Representative western blots of the expression levels of caspase 3 (Casp 3) and cytochrome c (Cyt c) in the PBS- and VEGF-treated fat grafts at the end of the 15-week study period.

The extent of apoptosis and the MVD observed in the PBS-treated fat grafts were the same in both the first and the second experiment. The MVD and the VEGF content in the VEGF-treated fat grafts were higher than, but not statistically different from, those in the PBS-treated fat grafts (Figures 5A and 5B). There was unorganized vessel formation and perivascular hemorrhage in the VEGF-treated fat grafts. The extent of apoptosis in the VEGF-treated fat grafts was greater than that observed in the PBS-treated fat grafts (Figure 5C). There were no statistical differences in the expression levels of caspase 3 and cytochrome c in the PBS-treated and in the VEGF-treated fat grafts (Figure 5D).

### The effect of EPO on endothelial cell proliferation and tube formation on matrigel

**Figure 6:**
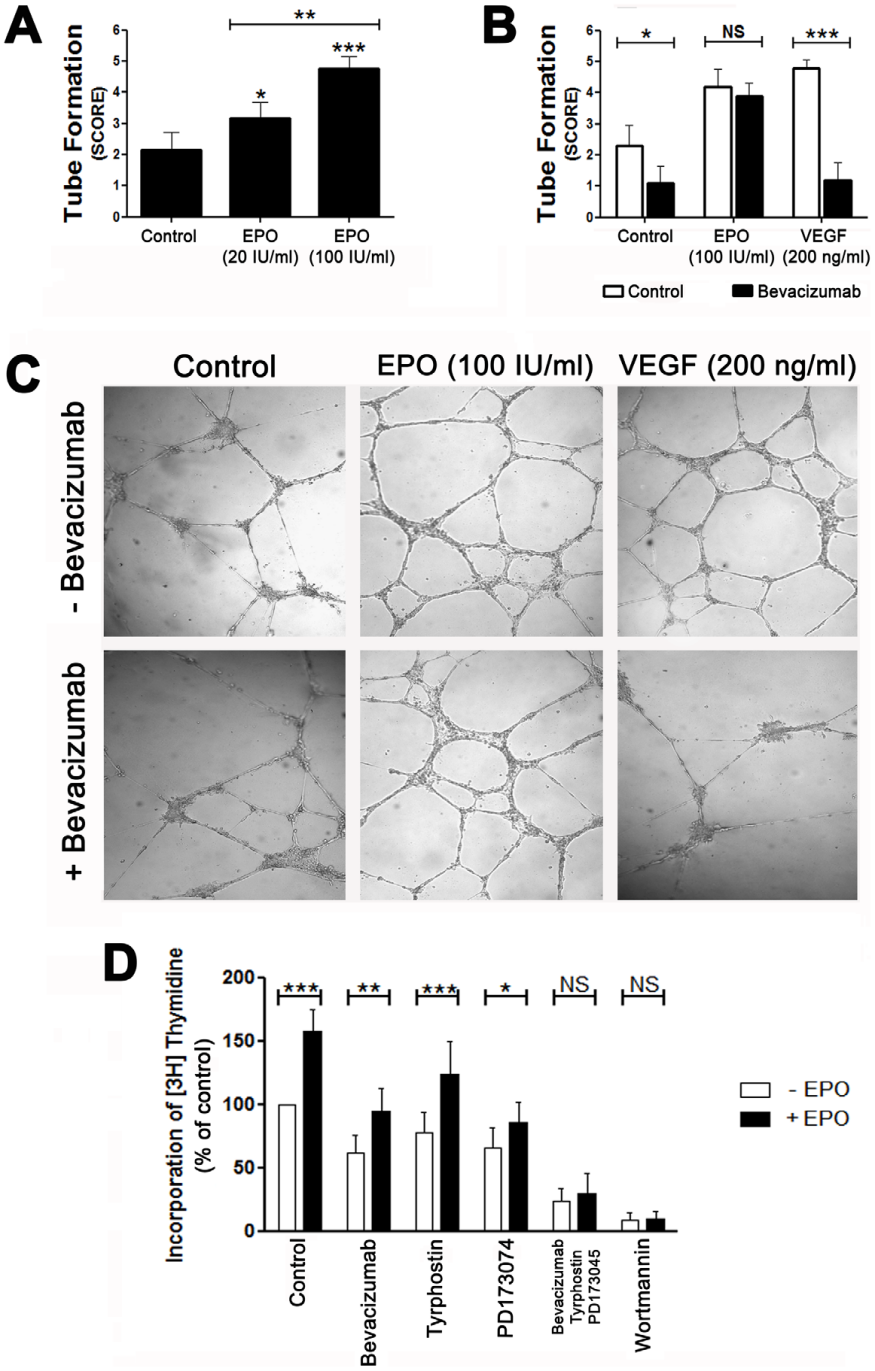
Effect of EPO on HUVEC proliferation and tube formation on matrigel. HUVECs were treated with either 20 IU/ml or 100 IU/ml and either 100 IU/ml EPO or 200 ng/ 100μl VEGF in the absence or presence of 0.25 mg/ml bevacizumab for 48 hours after plating the cells on matrigel. The extent of HUVEC tube formation on matrigel was assessed after 24 hours under a light microscope at 10× magnification. The tubular structures were graded semiquantitatively on a scale of 0 to 5 by evaluation of the relative presence and stages of formation of tubes on the matrigel: 0 =  well separated individual cells, 1 =  cells had begun to migrate and align themselves, 2 =  visible capillary tubes and no sprouting, 3 =  visible sprouting of new capillary tubes, 4 =  early formation of closed polygons, 5 =  development of complex mesh-like structures. (A) Each bar represents the mean grade of tube formation ± SD in the matrigel. *P<0.05, **P<0.01 and ***P<0.001. (B) The white bars are the mean grade of tube formation ± SD in the matrigel of untreated HUVECs, VEGF- and EPO-treated HUVECs. The black bars represent the mean grade of tube formation ± SD in the matrigel of untreated HUVECs VEGF- and EPO-treated HUVECs that were exposed to bevacizumab. *P<0.05 and ***P<0.001, for the difference between HUVECs that were or were not exposed to bevacizumab. NS  =  not significantly different. (C) From top to bottom: representative micrograph of untreated HUVECs on matrigel, VEGF- and EPO-treated HUVECs after 24 hours of plating with (+) or without (−) bevacizumab. (D) Cultured HUVECs were treated with or without 100 IU/ml EPO in the presence of either bevacizumab, PD173074, or tyrphostin, a combination of bevacizumab, PD173074 and tyrphostin, or in the presence of wortmannin. Proliferation of HUVECs was measured by incorporation of [^3^H]-thymidine to DNA. Duplicate cell counts were averaged for 3 experiments and the data were expressed as the percentage of control. *P<0.05, **P<0.01 and ***P<0.001 for the difference between untreated, or EPO- treated HUVECs that were exposed to bevacizumab, PD173074, tyrphostin or wortmannin. NS  =  not significantly different.

VEGF significantly increased HUVEC tube formation and EPO increased HUVEC tube formation in a dose-dependent manner (Figure 6A). Tube formation was substantially reduced in VEGF + bevacizumab-treated HUVECs, but not in the EPO + bevacizumab-treated HUVECs (Figures 6B and 6C). The VEGF inhibitor, bFGF inhibitor and PDGF inhibitor each reduced HUVEC proliferation significantly, whereas either a combination of the 3 inhibitors together or wortmannin alone abolished HUVEC proliferation. EPO normalized HUVEC proliferation in the presence of any of the inhibitors, but had no effect on HUVEC proliferation in the presence of a combination of the 3 inhibitors together or in the presence of wortmannin alone (Figure 6D).

## Discussion

The main finding of our study is that the decrease in the weight and volume of EPO-treated human fat grafts was smaller than the decrease that was observed in VEGF- and PBS-treated human fat grafts in immunologically-compromised nude mice. Treatment of the fat grafts with EPO (a) increased the expression levels of various angiogenic factors, induced the expression of cell survival factors such as PKB, and increased the extent of MVD, (b) increased fat cell survival and (c) reduced the extent of inflammatory response and fat cell apoptosis in a dose-dependent manner. The histological assessments of the harvested fat grafts showed that EPO treatment led to better fat tissue integration with less cysts, fibrosis, and inflammatory cell infiltration compared to the PBS- and VEGF-treated fat grafts. From these results, we concluded that treatment of fat grafts with EPO improves the fat graft's integration into the surrounding tissues and its long-term survival following fat transplantation. Our data suggests that EPO-induced angiogenesis in the transplanted graft occurs due to stimulation of a cluster of angiogenic factors that include VEGF, bFGF, PDGF-BB, MMP-2, and IGF-1 that has been shown to increase the survival of grafted fat cells [32]. These findings are in agreement with those of Pallua and colleagues, who reported that VEGF, bFGF, PDGF-BB, and IGF-1 are all required for promoting fat cell viability and adipogenesis [33].

Vascularization is essential for graft survival. After autologous fat transplantation, the increased resorption and the inability of a fat graft to survive in the recipient is associated with reduced fat tissue vascularization, and increased apoptosis of fat cells in the graft [20], [21], [22]. VEGF is a known potent angiogenic factor that influences endothelial proliferation, migration and viability and induces angiogenesis of adipose tissue after transplantation [34].VEGF gene therapy in fat grafts can induce angiogenesis and enhance fat cell survival and viability [21]. Recently, Lu and colleagues demonstrated in an elegant study, that modified adipose-derived stem cells that overexpressed VEGF can enhance the survival and quality of transplanted fat tissue through an angiogenesis-dependent mechanism [26]. In our study, we demonstrated that exogenous VEGF treatment of fat grafts has no effect on their weight and volume compared to the same parameters in PBS-treated fat grafts. We also observed that the MVD of the VEGF-treated fat grafts was modestly higher than that of the PBS-treated fat grafts. In addition, we found that the extent of apoptosis in these VEGF-treated fat grafts was not different from that in the PBS-treated fat grafts, and this could probably indicate that VEGF does not exert an anti-apoptotic effect in the fat grafts. The process of angiogenesis involves a harmonized interplay between various angiogenic factors that include growth factors such as bFGF, VEGF, PDGF-BB, and proteases such as MMPs that digest constituents of the extracellular matrix that impede angiogenesis. These factors act synergistically in order to improve the survival of adipose tissue after fat transplantation [23], [24]. Therefore, the therapeutic use of one of these angiogenic factors, even one as potent as VEGF, may not be sufficient to promote angiogenesis for enhancing fat tissue viability and survival. Contrary to the findings of Yi [21], Lei [25], Lu [26] and their colleagues who found that either gene therapy with VEGF, or adipose derived-stem cell therapy that overexpressed VEGF, can enhance fat cell viability and survival, we are of the opinion that the angiogenic actions of exogenous VEGF may not be adequate to elicit an appropriate angiogenic response in transplanted fat tissue. Indeed, the increased VEGFR-2 expression in the EPO-treated fat grafts that was observed in our study implies that EPO-induced endogenous VEGF secretion in the fat grafts might be more effective than exogenous VEGF administration.

Nakano and colleagues demonstrated that EPO treatment increases VEGF expression and promotes angiogenesis in peripheral ischemic tissues in mice [10]. We recently reported that topical EPO treatment induces VEGF secretion and angiogenesis in excisional wounds in diabetic rats [35]. In the current study, we showed that treatment of fat grafts with EPO increased VEGF, bFGF, IGF-1, PDGF-BB and MMP-2 contents, as well as MVD in the fat grafts. We also observed that, similarly to VEGF, EPO increased HUVEC tube formation on matrigel, thereby confirming that EPO has angiogenic activity. Interestingly, bevacizumab abolished VEGF-induced tube formation, but not EPO-induced tube formation. This result suggests that the angiogenic activity of EPO on HUVECs is indirect, and could be mediated by stimulating other growth factors, such as bFGF and PDGF-BB, and proteases such MMP-2. Furthermore, EPO normalized *in vitro* HUVEC proliferation in the presence of a single growth factor inhibitor such as bevacizumab (VEGF inhibitor), PD173074 (bFGF inhibitor) or tyrphostin (PDGF inhibitor), strengthening our claim that the use of one growth factor for fat tissue vascularization might not be adequate. On the other hand, EPO has no effect on the proliferation of HUVECs that were exposed to the above mentioned inhibitors simultaneously, supporting the idea that secretion of a cluster of growth factors accounts, at least, to one of the underlying mechanisms of EPO action on fat graft vascularization. In addition, EPO increased PKB expression and activity in the fat grafts, and PKB is critical for the cellular pathway of a broad spectrum of growth factors. Nevertheless, EPO has no effect on the proliferation of HUVECs that were exposed to wortmannin which inhibits the phosphorylation of phosphatidylinositol 3-kinases (PI 3-K) and subsequently the phosphorylation of PKB, confirming that EPO stimulates the secretion of multiple growth factors, at least partially, through the PI 3-K/PKB cellular pathway. In light of our results, we attribute the beneficial effects of EPO on the improved viability of fat grafts, partly, to this action on angiogenesis in the fat grafts.

Nishimura and colleagues reported that the sustained volume loss of fat grafts, even in those that are vascularized, is due to fat cell apoptosis [22]. We observed that the inflammatory response in the PBS-treated fat grafts was greater than the inflammatory response in the EPO-treated fat grafts. This increased inflammation can be attributed to leukocyte and macrophage infiltration, and to increased cytokine secretion in the fat graft. We found that the extent of apoptosis in the EPO-treated fat grafts decreased in a dose-dependent manner. These findings are not surprising since EPO has well-known anti-apoptotic properties, and is an anti-inflammatory cytokine [15]. Accordingly, we concluded that EPO decreases the rate of fat resorption by directly decreasing the extent of apoptosis in fat cells and/or indirectly by suppressing the inflammatory response that ensues after fat grafting.

Aspirated fat tissue that is used for autologous fat transplantation is devoid of blood microvessels because these microvessels are destroyed during the aspiration, and removed during processing prior to its injection. Therefore, the fat tissue that is injected into a recipient is considered to be an ischemic fat cell mass. During the early period following transplantation, the fat transplant exists under hypoxic and hyponutritional conditions. Should revascularization fail to be initiated in this early period, apoptosis ensues and results in late fat cell degeneration and fat resorption [22]. In this study, we provoked angiogenesis at the time of fat injection, and for the 18 days after transplantation, by repeated injections of EPO into the fat graft. By doing this, we hoped to induce angiogenesis in the transplanted fat tissue while it is still an ischemic cell mass, in order to promote fat cell survival by increasing the delivery of oxygen and nutrients. At the same time, we thought that this treatment would protect the transplanted fat cell mass from early degeneration, and delay and/or prevent fat cell apoptosis. As already noted, EPO exerts an anti-apoptotic action on fat cells because EPO decreases the extent of DNA fragmentation, caspase-3 activity and cytochrome c in the fat grafts. This anti-apoptotic action might be obtained due to a direct effect of EPO on fat cell apoptosis, and/or an indirect effect by promoting fat graft vascularization. Nevertheless, treatment of the fat grafts with exogenous VEGF did not alter the extent of apoptosis in the fat grafts, although it had modestly increased the vascularization in the fat grafts compared to control fat grafts.

The overall clinical experience on the use of growth factors and cytokines to reduce the rate of fat resorption by increasing fat graft vascularization has not been encouraging [21], [36], [37]. We found that fat graft treatment by EPO improves the survival of a human fat graft in nude mice since EPO not only increased angiogenesis, but also reduced the inflammatory response and fat cell apoptosis. EPO can account for some use-limiting adverse effects, as it may promote hypertension, retinopathy, neurotoxicity and thrombotic events when it is used in the repetitive and large doses that are required for adequate tissue protection. It may also lead to an increased risk of spread of tumor growth due to its effect on angiogenesis, as has been observed particularly in patients with chronic diseases and in patients with cancer [38]. Nevertheless, EPO has been safely used in humans for many years for treating anemia, and in trials that tested EPO as a neuroprotective/neuroregenerative agent [39]. The production of EPO by means of recombinant techniques and its availability in various competent recombinant forms, make EPO an economical drug, conferring it as a potential candidate for enhancing fat transplantation without increasing considerably the cost of the procedure.

In conclusion, the failure of exogenous VEGF to stimulate adequate angiogenesis and to prevent apoptosis in fat grafts strengthens our hypothesis that fat cell survival and viability depend on the action of a cluster of angiogenic factors as well as on prevention of fat cell apoptosis, a process which can be improved by promoting them. We found that EPO treatment of transplanted fat acts through these two mechanisms, and can improve fat graft integration and its long-term survival in immunologically-compromised nude mice. Based on our results, we propose that EPO treatment can significantly improve the efficacy of human autologous fat transplantation for soft tissue filling and augmentation. To the best of our knowledge, the results of this study are the first to demonstrate the effect of EPO on fat resorption. Further studies in animals and humans are now needed in order to validate our data.
